# Book Reviews

**Published:** 1894-08

**Authors:** 


### Book Reviews.

AN INTERNATIONAL SYSTEM OF ELECTRO-THERAPEUTICS, for Students, General Practitioners and Specialists. By HORATIO R. BIGELOW, M. D., and Thirty-eight Associate Editors. Thoroughly illustrated. In one large royal octavo volume. 1,160 pages; extra cloth, $6.00 net; sheep, $7.00 net; half-Russia, $7.50 net. Philadelphia: The F. A. Davis Co., Publishers, 1914 and 1916 Cherry street. 1894.

Everyone familiar with the literature of medicine during the last few years, must have become impressed with the fact that there has been a remarkable attempt on the part of a coterie of enthusiasts to force electricity into a prominence which no other alleged therapeutic agent possesses. One need not go far to find a reason for this. In the first place there is something about the mysterious that always appeals to certain minds. We remember an old dictionary, in defining mesmerism, stated it was "practised by the weakest, meanest, sometimes the most infamous of mankind." This will not apply to the electromaniacs of the present day, many of whom are thoroughly conscientious men and we hope all of them are honest in their purposes.

So little is known about electricity as a therapeutic agent, that the temptation is very great to appeal to it when other remedies fail, in the hope that by some magic, unseen and unknown influence it may effect a cure. It is an agent that is applied in all fashions and ways, from the simple toy and useless plaything called a pocket battery to the imposing static machine. With how much science these several machines are used depends largely, we may say almost entirely, upon the man who applies them. But a factor in their successful employment is also to be found in the patient who receives this subtle and mysterious dosage. If faith goes a large way toward effecting cure with any agent, it certainly has an opportunity to work the marvelous with electricity.

These remarks are not intended to apply to certain well-defined and known principles of its application, as instanced in the electro-cautery, and, further, in its diagnostic uses in certain nervous diseases. But we speak more especially of its employment in the thousand and one conditions, all the way from sore nipples to fibroid tumors of the uterus.

A work of the magnitude of the present one which aims to be encyclopedic in character challenges criticism, first, from the men who create it,—editor and contributors—and, second, from the material that they present. The editor-in-chief of this system has been a frequent contributor to journalistic literature, but we are not aware that he has substantially advanced or improved the science of medicine by his writings. While many of the associate editors have achieved prominence, a number of them are quite unknown, and several of them are more or less visionary or enthusiastic in their advocacy of electricity as a therapeutic measure applicable to almost every known malady.

The introduction to the volume is written by William J. Herdman, of Ann Arbor, and contains, perhaps, all that need be said in an introductory chapter. We note, on page xxvi., that the "practicing" physician needs not to be a pharmacist, in order that he may "skillfully" administer his remedies, etc., which is a method of spelling not usually adopted in the present day. Professor Duff's section on electro-physics is a most instructive exposition of the subject, as is also the next section on animal electricity, written by Wesley Mills.

The faradic current, electro-magnetism, electro-massage and instruments, forms the subject of the next section, and is written by Engelmann, of St. Louis, of whom there is none more competent. Cataphoresis is interestingly handled by Peterson, of New York, who is one of the pioneers in this method, and whose experiments have been published in detail in the journals. Ectopic gestation forms the subject of a section written by Carter S. Cole and George W. Jarman, and revised by Egbert H. Grandin. Our opinion in reference to the employment of electricity in this condition, has often been expressed before in the columns of this journal, hence need not be repeated in detail here. We do not believe that electricity has any place here. This is essentially a surgical condition, and should be treated surgically, with all that the term implies. In an editorial review on Mr. Tait's lectures on Ectopic Pregnancy, on page 462 *et seq.*, Volume XXVIII., will be found a full elaboration of the subject, to which we refer those of our readers who may be interested.

Facial blemishes forms a brief but interesting section by

Henrietta P. Johnson. Electricity has a special application here, and we should be pleased to see the subject enlarged upon. Diseases of the nose, naso-pharynx, pharynx and larynx form another field where electricity is especially applicable, and is ably considered by Charles E. Sajous, the well-known editor of the Annual of the Universal Medical Sciences. Electro-thermal surgery—that is, the galvano-cautery considered with reference to its surgical uses—is ably considered by the veteran John Byrne, of Brooklyn, than whom there is no more competent authority. It must be confessed, however, that Byrne's most famous operation—amputation of the lower segment of the cancerous uterus—has been almost wholly supplanted by its total ablation per vaginam, known as vaginal hysterectomy. One of the most curious chapters in the book is contributed by Mary Putnam Jacobi, under the title of electricity in diseases of childhood. There is hardly any of the diseases treated of in this chapter by the distinguished author that do not belong to advanced youth or adult age. Yet it is learnedly written and is really one of the most interesting and instructive chapters in the book. We are at a loss to understand why the final section, that treats of adhesions in the acute and chronic inflammatory disorders of the female pelvis, has been presented. It is written by J. M. Baldy, of Philadelphia, who is well known as a surgeon of distinction, and who has little use for electricity, especially in the conditions of which this subject treats. He goes into considerable pathological detail in this chapter, but has little to say in regard to electricity. He sums up his opinion as follows:

> To speak dogmatically, we may say (1) that the electrical treatment finds its greatest usefulness in cases of simple, acute inflammation with exudation of plastic lymph; (2) that in chronic processes in which an acute inflammation with exudation has supervened, the latter is amenable to the treatment in just the same manner and to the same extent as a simple case without chronic disease; (3) that chronic, fully-organized adhesions are rarely benefited, and may be deleteriously influenced; and (4) that friable, unhealthy, degenerated deposits are not at all benefited by this mode of treatment.

The chapters mentioned represent whatever there is of excellence in the work, though in some of the others there are paragraphs of interest. An examination of the treatise cannot fail to impress the reader with the fact that an attempt is making to treat almost every disease of the economy, from the most trifling to the gravest, with electricity. And what is still more impressive is that enthusiasts are claiming cures in the majority of instances.

The American Electro-Therapeutic Association is an outgrowth of this enthusiasm, and has as much reason for existence as would an association formed on the basis of any other single therapeutic measure. This treatise is a natural sequence to that association, and we presume that its members will regard it with special favor as, indeed, many of them are contributors to its pages.

We observe that this system is intended for students, general practitioners and specialists, and, as these three classes cover the entire field of medicine, if they should all conclude to purchase the book it would exhaust the first edition speedily.

There is the usual display of illustrations, in which batteries and electrodes figure without number; the articles of Dr. Mills and Dr. Byrne, however, contain cuts of another nature. The volume is well printed, and, no doubt, will command ready sale.

---

TUMORS—INNOCENT AND MALIGNANT. Their Clinical Features and Appropriate Treatment. By J. BLAND SUTTON, F. R. C. S., Assistant Surgeon to the Middlesex Hospital, London. In one 8vo volume of 526 pages, with 250 engravings and nine full-page plates. Cloth, $4.50. Philadelphia: Lea Brothers & Co., Publishers. 1894.

A new work on one of the most important pathological subjects, by one of the foremost pathologists and surgeons of London, must certainly attract considerable attention and be carefully perused by physicians generally. For many years the author has been collecting material and data for this work, and has made very interesting reading of an otherwise difficult and perplexing subject.

Virchow's classification is adopted with a few changes, notably the exclusion of the infective granulomata and the inclusion of the myomata, neuromata and angiomata under the histoid or connective tissue group. Sutton thus arranges tumors: (1) connective tissue tumors; (2) epithelial tumors; (3) dermoids; (4) cysts. Each group has several genera, each genus has one or more species, and of each species there may be one or more varieties. The connective tissue group is described at some length, notably the sarcomata, and each genus is described in the author's peerless style as to appearance, location, clinical features and treatment. Many rare and unusual cases and specimens, especially those preserved in the museum of the Royal College of Surgeons of London, are nicely illustrated.

Under the head of epithelial tumors are classed the papilomata, epitheliomata, adenomata and carcinomata—the latter only being regarded as cancers. Considerable attention is given the psammomata, occurring in the brain and spinal membranes, while the cholesteatomata are dismissed with the remark that it is urgently necessary to drop the name.

An interesting chapter follows the psammomata, on cutaneous horns, with illustrations of exceptional cases occurring in man and in the lower animals. These more properly come under the head of freaks. The carcinomata and adenomata are treated together, both constructed upon the type of secreting glandular tissue, but differing in the regularity of their structures. The various organs of the body prone to carcinomatous encroachment are discussed and treatment is advised. In the majority of cases the knife is recommended, and many statistical tables are presented giving the result of operative procedures.

The chapters on dermoids are very interesting, especially that devoted to the branchial clefts. These are congenital fistulous openings on the side of the neck, and have been cited by evolutionists as belonging to the anatomical defects which link the human species with the finny species. They have been discovered in man, horses, sheep, pigs and chicks.

The chapter on teratoma contains malformations, parasitic fetuses, supernumary limbs, etc. The portrait of Laloo, exhibited a short time ago in one of Buffalo's amusement halls, is here reproduced.

The remaining chapters are devoted to a very careful study of cysts, of which the author describes and generalizes retention cysts, tubulo-cysts, hydroceles, glaced cysts, and the false cysts, as diverticula, bursal, neural cysts, and parasites.

One cannot close this book without a feeling of gratitude for the completeness and simplicity with which each subject is treated. It serves very well as an encyclopedia on tumors and a key to the detection and classification of unusual forms of tissue development.

The publishers have aided materially in making the volume an unqualified success. W. C. K.

---

Biography of Eminent American Physicians and Surgeons. Edited by R. French Stone, M. D., author of Elements of Modern Medicine, Surgeon-General National Guard, State of Indiana; Consulting Physician to the Indianapolis City Hospital and Dispensary; Ex-President of the Marion County Medical Society; Member of the Indiana State Medical Society and American Medical Association; formerly Professor of Materia Medica, Therapeutics and Clinical Medicine in the Central College of Physicians and Surgeons, etc. Illustrated with hundreds of fine photo-engraved portraits and autographs. Published by Carlon & Hollenbeck, of Indianapolis, in one large 8vo volume, containing 751 double-columned printed pages, and to be sold by subscription only. Price, in cloth, $8; leather, $9, and half morocco, $10, sent c. o. d., express charges prepaid.

But few attempts have been made to publish biographical cyclopedias of American physicians. Dr. James Thatcher, of Massachusetts, was first to perform this duty, in a work published in 1828. Next came the work edited by Dr. Stephen W. Williams, published in 1845, which was supplementary to that of Dr. Thatcher. Dr. Samuel D. Gross was the third to enter this field, and the work he edited was published in 1861, while the fourth was published in 1878 by Dr. William B. Atkinson. The fifth venture in this line is the work before us, which is offered to the profession as the most complete encyclopedic medical biography yet written.

It contains a preface and an introductory chapter, both of much interest. The biographical sketches are arranged alphabetically, many of which are carefully written and enter into great detail. Some of them, however, are carelessly prepared and give fulsome praise in general or in special, which is out of place in a work of this kind. This cannot be charged against the editor as a fault, but is due rather to the contributor--the subject of the sketch or an enthusiastic friend.

In our opinion, a biographical sketch in a work of this kind should be a plain recital of facts, beginning with the birth of the subject and giving all the prominent data of his life down to the present, without any elaboration or multiplication of adjectives. It is gratifying to know that most of the biographies in this work are free from the objectionable features referred to.

The chief criticism of this book we are inclined to regard as lying in its illustrations. These are reproductions from photographs, or other likenesses, and, while some of them are excellent, many of them are artistically deficient and do but sad justice to the subject that they are intended to illustrate.

The sketches of some of the historic characters in medicine, now dead, are of a high order and serve to enhance the value of the work. Those of David Hosack and Benjamin Rush are exceptionally fine and invaluable.

The value of this work is beyond question. It is needed for reference in the office of every progressive physician, while teachers, authors, editors and scientific writers cannot afford to be without it. All medical libraries, too, should make haste to obtain it, while general libraries must assuredly place it upon their shelves.

---

An Illustrated Dictionary of Medicine, Biology and Allied Sciences, including the Pronunciation, Accentuation, Derivation and Definition of the Terms used in Medicine, Anatomy, Surgery, Obstetrics, Gynecology, Therapeutics, Materia Medica, Pathology, Dermatology, Pediatrics, Ophthalmology, Otology, Laryngology, Physiology, Neurology, Histology, Toxicology, Dietetics, Legal Medicine, Psychology, Climatology, etc., etc., and the various Sciences closely related to Medicine, Bacteriology, Parasitology, Microscopy, Botany, Zoölogy, Dentistry, Pharmacy, Chemistry, Hygiene, Electricity, Veterinary Medicine, etc. By George M. Gould, A. M., M. D., Author of The Students' Medical Dictionary, 12,000 Medical Words Pronounced and Defined, The Meaning and the Method of Life; Editor of *The Medical News;* President, 1893-1894, American Academy of Medicine; one of the Ophthalmologists of the Philadelphia Hospital. Based upon recent scientific literature. Double-columned quarto, pp. xvi.—1633. Philadelphia: P. Blakiston, Son & Co., 1012 Walnut street. 1894. Price in sheep or half-Morocco, $10 net; half-Russia, thumb index, $12 net.

This may be fittingly termed the period of the renaissance of medical word-book making. For forty years beginning with 1839 the professional public chiefly depended upon Dunglison's Medical Dictionary for an explanation of words and phrases used in medicine. This work grew in its fourth edition to contain about 770 octavo pages, and claimed, not without justice, to be a work "in which the student may search without disappointment for every term that has been legitimated in the nomenclature of the science."

It is but a little over ten years ago that medical lexicographers, recognizing the increasing demands of the science, began to bestir themselves in the preparation of new dictionaries that should more adequately cope with the rapidly increasing nomenclature of medicine. Since then there has sprung up somewhat suddenly a great excitement, so to speak, similar to that which follows upon the discovery of a new bonanza, and medical lexicographers have literally had the floor for a decade, more or less.

The work of Gould, which is before us, challenges at the outset admiration and respect both for its handsome appearance and the vastness of its contents. In the first place the type selected is of a size and kind that does not tire the eye ; the word to be defined is displayed and the definitions are given in smaller faced letters. The origin of words is given whenever necessary, while their pronunciation and orthography are fully elaborated. We are more than gratified to observe that diphthongs have been eliminated wherever practicable, and the practicability of this change is constantly increasing. The table of abbreviations is a large one and needs to be carefully studied lest confusion arise.

An excellent arrangement is to give, as Gould has done, a table of prefixes and suffixes used in medical terms. Besides these useful tables that precede the dictionary proper, there are many others interspersed throughout the text in their appropriate places that serve to aid the student—we use the word in its most comprehensive sense—in an understanding of the subject upon which information is sought. For example, beginning on page 217 and running to 229 inclusive, is an illustrated table of bones, that serves as a most convenient aid to the memory with reference to this important anatomical subject, and we should advise every author of works on human anatomy to follow Gould's example and publish a similar table. We might say a word, too, in commendation of all the anatomical tables throughout the book if it were necessary. There are, however, some tables that we think might have been omitted without doing harm to the work and that would thus have reduced its size. We might instance an example to illustrate this point in the table of conspectus of pigments. The suture illustrations are valuable because no one can understand the application of a suture alone by its description.

We cannot go into an analysis of the definition of words within the space here allotted us, but we may affirm that Gould has given for the most part the clearest and most concise definitions that are consistent with the purposes of his work ; and while one might here and there wish that he had either said more or fewer words on a particular subject or subjects, we cannot but believe that the profession, by and large, will accept this dictionary as the best single volume lexicography extant, and it must of necessity find its way to the book shelves of every scientific man.

---

A Text-Book on Diseases of the Eye. By Henry D. Noyes, A. M., M. D., Professor of Ophthalmology and Otology in Bellevue Hospital Medical College, Executive Surgeon to the New York Eye and Ear Infirmary, etc., etc. Second and revised edition, illustrated by five chromo-lithographic plates, ten plates in black and colors and 269 wood-engravings. Pp. xviii.—816. New York: Wm. Wood & Co. Price in cloth, $6.00 ; leather, $7.00. 1894.

Text-books indigenous to America have never been very numerous on diseases of the eye. In 1823, George Frick, M. D., of Baltimore, published a brief "treatise ;" in 1837, S. Little, Jr., M. D., surgeon to Wills Hospital, of Philadelphia, issued a concise but well written "manual," which passed through a second and much enlarged edition in 1848 ; in 1832, John Mason Gibson, M. D., of Baltimore, wrote a very poor "condensation ;" in 1850, Henry Howard, M. R. C. S. L., of Montreal, Canada, presented a good compilation ; in 1862, Henry W. Williams, M. D., of Boston, gave to the profession a "guide" which has passed through several editions, and to the present time has held its place as most practical and trustworthy. These were followed later by Noyes (Woods Library, 1881), Schell (Philadelphia, 1881), Mittendorf (New York, 1882), Alt (St. Louis, 1884), Hansell & Bell (Philadelphia, 1892), DeSchweintz (Philadelphia, 1892), Norris & Oliver (Philadelphia, 1893), and some minor compilations.

This list is not a long one, and among it very few authors can be found who have left distinct marks of their own opinions and conclusions. We do not detract from the value of the masterly treatise of Norris & Oliver, or the excellent compilations of DeSchweintz and Mittendorf, or the very safe and practical "guide" of Williams, when we say that Noyes's text-book stands first and above all the others in representing the author's personal views. Dr. Noyes is a man of vast experience. He is an original thinker and close observer. He respectfully considers the conclusions of others and tests their validity whenever practicable. Out of this rich storehouse of the experience of himself and others, he has elaborated this text-book.

This edition may be considered in reality as the third, although it is known as the second, the first being the volume contributed to "Wood's Library." It has grown and improved with each new issue. It is much better now than ever before, as it has undergone thorough revision, and every branch of the subject has been brought up to date. The chapters dealing with refractive and muscular anomalies, glaucoma, granular conjunctivitis and the optic nerve, have been especially amplified. Under the heads of Asthenopia, and Binocular Vision and its Disturbances, will be found clear statements of the results of the author's experiences regarding muscular errors and muscular therapeutics, subjects so engrossing to the attention of ophthalmologists at the present time. The author's views are here somewhat conservative, and may be questioned by some ; but, based as they are upon a study extending over many years, and upon thousands of cases of muscular iniquilibrium, they cannot be ignored.

This treatise is most complete in every respect. Scarcely a subject in ophthalmology can be recalled that is not here treated. Not only are the author's views presented, but constant reference is also made to those of others, and the sources of information are indicated either in the body of the text or in foot-notes. Notwithstanding the fact that a few literary blemishes mar the "finish" of the work, it is a credit to American medical literature and to scientific ophthalmology. In short, the work emanates from an independent and judicious mind; its opinions are grounded in an immense and well-weighed experience ; it is complete ; it is conservative, but not antiquated ; it is safe.

The commendations which the JOURNAL gave in June, 1890, to the previous edition can but be emphasized now. A. A. H.

———

GONORRHEA : Being the Translation of Blenorrhea of the Sexual Organs and Its Complications. By DR. ERNEST FINGER, Docent at the University of Vienna. One volume, of 330 pages, octavo, illustrated by numerous wood engravings, and by seven chromo-lithographic plates. Third revised and enlarged edition. Bound in muslin, gold lettered, $3.00. New York : William Wood & Company, 1894.

Since the investigations of Neisser, who first pronounced gonorrhea an infectious disease, an increased interest has attached to its study and new responsibilities pertained to its treatment. We have on several occasions in the columns of the JOURNAL called attention to the dangers that lurk in a neglected or uncured gonorrhea. Since the discovery of the gonococcus by Neisser and the warning of Noeggerath regarding the dangers of infection from latent gonorrhea, the teachings of the schools have been entirely reconstructed and venereal practice has changed front.

Prior to the time referred to, syphilis was regarded as the most important and dangerous venereal disease. Then clap was regarded as a mere trifle that a few days dosing with cubebs and copaiba and a number of injections of a solution of sulphate of zinc would assuredly cure. Or, if in a few instances a gleet remained this was regarded as non-infectious and of little or no consequence. Even now we see paraded in the newspapers advertisements of big G, in which it is announced as a sure cure for this distressing and dangerous malady.

When one contemplates the myriads of innocent women who have been infected through this abominable doctrine and practice, and whose lives when not destroyed have been made utterly miserable, whose pus tubes and aching ovaries have told a tale of woe that ought to melt the stoutest heart, we feel as though indictment and imprisonment would be a light punishment for doctors who thus treat gonorrhea and for newspaper proprietors who admit big G to its advertising pages.

Finger attacks this disease from the most modern standpoint. He gives its history and etiology in two introductory chapters, and then considers blenorrhea in the male and its complications, beginning with urethral blenorrhea, on which he offers at the outset timely anatomical and physiological remarks. Under acute urethritis the real consideration of the subject is reached, and in this chapter infection, acute anterior and posterior urethritis, together with diagnosis, prognosis and treatment, are exhaustively considered and most scientifically handled. But we think the chapter on chronic urethritis is the most important in the book—the most important because it is apt to be the most neglected; and, again, because its neglect leads to such serious consequences; and still again, the most important, because here the truly earnest physician will find ways and methods for the cure of this obstinate and subtle malady. Complications in the male and in the female are considered in the next two chapters, and, finally, a chapter is devoted to complications of blenorrhea in both sexes.

It has been reserved for Finger to make the first systematic anatomical examinations of chronic urethritis, and he has given in well prepared colored plates an excellent picturing of the subject. Every general practitioner, especially those in the country, should possess this book. It is an intelligent, safe and simple setting forth of the subject, and this third edition brings it down to the present date.

---

A MANUAL OF PRACTICAL OBSTETRICS. By EDWARD P. DAVIS, A. M., M. D., Professor of Obstetrics and Diseases of Infancy in the Philadelphia Polyclinic; Clinical Professor of Pediatrics in the Woman's Medical College; Clinical Lecturer on Obstetrics and Gynecology in the Jefferson Medical College, etc., etc. Second edition, revised and enlarged. With 134 illustrations and sixteen full-page plates, several of which are colored. Small 8vo, pp. xii.—351. Price, $2.50. P. Blakiston, Son & Co., 1012 Walnut street. 1894.

The importance attached to the study of obstetrics is accentuated very much when a second edition of a manual like this one is demanded so soon after the original appearance of the work. No matter how many elaborate treatises one has in his library he must needs have manuals as well, and of all the obstetric manuals that we have seen, this one approaches nearer to the ideal than any other.

The illustrations are of an intelligent order, some of which are new. The present edition considers symphyseotomy in a clear but brief manner. So much attention is attached to this subject in view of its recent revival as an alternative in narrow pelves, that we could wish the author had been a little more elaborate in its consideration.

We are glad to notice that Dr. Davis adopts the term puerperal sepsis in preference to the old and misleading one of puerperal fever. We commend this manual as admirably filling the place for which the author has constructed it.

---

A Treatise of Headache and Neuralgia, including Spinal Irritation and a Disquisition on Normal and Morbid Sleep. By J. Leonard Corning, M. A., M. D. Consultant in Nervous Diseases to St. Francis' Hospital; Fellow of the New York Academy of Medicine; Member of the New York Neurological Society, etc. With an Appendix. Eye Strain, Cause of Headache. By David Webster, M. D., Professor of Ophthalmology in the New York Polyclinic; Surgeon to the Manhattan Eye and Ear Hospital, etc., etc. Illustrated. Third edition. Small 8vo, pp. 275. New York: E. B. Treat, 5 Cooper Union. London: H. K. Lewis, 136 Grower street. 1894. Price, $2.75.

The fact that a third edition is called for by the profession shows that the author has succeeded in supplying a work adapted to the needs of the busy practitioner.

This edition differs from the previous one in the addition of an appendix on eye strain as a cause of headache, by David Webster, M. D. This considers headache due to refractive errors, impaired accommodation and insufficiency of the extrinsic ocular muscles. He merely presents clinical reports, but does not enter into argument. The book should be well received by the profession.

J. W. P.

---

Pain in its Neuro-Pathological, Diagnostic, Medico-Legal and Neuro-Therapeutic Relations. By J. Leonard Corning, A. M., M. D., Consultant in Nervous Diseases to St. Francis' Hospital, St. Mary's Hospital, the Hackensack Hospital, etc., etc. Illustrated. Small 8vo, pp. 328. Price, $1.75. Philadelphia: J. B. Lippincott Company. 1894.

The laconic title of this book strikingly reminds one of Mr. Hilton's classic—Rest and Pain. This author, however, limits his treatise to the neuro-pathological significance of pain as a symptom and then considers it in its diagnostic medico-legal and neuro-therapeutic relations. In other words, it may be asserted that this work is devoted to a consideration of the relation of pain to neurology.

In part first, consisting of nine chapters, the physiological, pathological and clinical relations of pain to disease are considered, while in part second, consisting of eleven chapters, is found an exposition of the special therapeutics of pain. The importance of learning to place a proper estimate upon the value of pain as a diagnostic symptom is very great, and it often becomes essential to treat pain as a symptom primarily, no matter how erroneous in theory this may seem. Pain is often so severe as to becloud the whole case and to prevent a proper reading of the phenomena of disease. Pain oftentimes masks the symptoms in a way to mislead or to precipitate error. Hence, it often becomes the first duty of the physician to address himself to the control of pain before venturing either diagnosis or intelligent treatment.

It is not to be understood by the foregoing remarks that we would advise an indiscriminate resort to opium or its preparations for the subjugation of pain. There are many other excellent methods of subduing this distressful symptom, and, speaking generally, they should be resorted to in preference to opiates. Nevertheless, in critical conditions, the depressing effect of pain is so great as to justify the most extreme measures in combatting.

The work before us furnishes an aid to the better understanding of pain in its relations to neurology. Though many will differ from the author in some of his observations, yet the book possesses interest and should command the patient consideration of the profession of medicine.

---

Practical Pathology. A Manual for Students and Practitioners. By G. Sims Woodhead, M. D., F. R. C. P., Ed.; Fellow of the Royal Society, Edinburgh; Director of the Laboratories of the Conjoint Board of the Royal Colleges of Physicians (Lond.) and Surgeons (Eng.); Pathologist to the Royal Infirmary, Edinburgh. With 195 colored illustrations. Third edition. Philadelphia: J. B. Lippincott Co. 1892.

The author of this excellent treatise begins at the very begining of pathological investigation, by describing in the first few chapters the instruments and arrangements necessary for conducting an autopsy, the measurements and weights of the various organs, methods of preservation and for hardening preparatory to microscopical examination. The different reagents and formulæ are carefully given and the technique explained in a painstaking manner.

Following these chapters on pathological histology come the inflammatory changes in the various organs and tissues, and the description of the tumors and animal parasites. Particularly interesting are the beautiful colored illustrations of the diseased tissues. These are not only pleasing to the eye, but are strikingly exact reproductions of what the microscope reveals. For instance, the illustration of a myxoma with picro-carmine stain, on page 539, is as natural as anything could be, and so throughout the work, this feature is equally well carried out. The descriptions of the various processes of disease are put in plain, careful language, avoiding ambiguity and redundancy.

On the whole, it is an excellent work for beginners in pathology and is to be recommended for the class-room and laboratory.

The publishers have done their best in making it a success, and a perusal of its pages will convince any one that they have succeeded admirably. W. C. K.

---

CLINICAL DIAGNOSIS. By ALBERT ABRAMS, M. D., Heidelberg; Professor of Pathology, Cooper Medical College, San Francisco; Pathologist to the City and County Hospital, San Francisco, etc., etc. Third edition, revised and enlarged. Illustrated. Small 8vo, pp. 273. Price, $2.75. New York: E. B. Treat, 5 Cooper Union. 1894.

This work on clinical diagnosis having passed to a third edition has become established in the mind of the profession as a useful guide to the study of this subject. This edition contains an amplification and revision of the former ones, and there is added to it many synoptic tables, as well as a chapter on insanity, together with a summary of recent methods of diagnosis. The study of clinical diagnosis is an important beginning to the understanding of disease. No intelligent therapeutics can be established without a thorough diagnosis, and a treatise on the subject may well constitute one of a series of medical classics as in the present instance.

---

ESSENTIALS OF PRACTICE OF PHARMACY, arranged in the form of Questions and Answers. Prepared especially for pharmaceutical students. Saunders' Question Compends, No. 18. Second edition, revised. By LUCIUS E. SAYRE, Ph. G., Professor of Pharmacy and Materia Medica of the School of Pharmacy of the University of Kansas. Duodecimo, pp. ix.—200. Price, $1.00. Philadelphia: W. B. Saunders, 925 Walnut street. 1894.

Of all the branches of medicine, chemistry and pharmacy, perhaps, are least understood, probably because they have not been thoroughly taught until of late. The medical student should know something of pharmacy, and this little book is well calculated to aid him in memorizing the essentials of that art. The student of pharmacy will find it invaluable.

---

TRANSACTIONS OF THE AMERICAN DERMATOLOGICAL ASSOCIATION at its seventeenth annual meeting, held at the Hotel Pfister, Milwaukee, Wis., on the 5th and 6th of September, 1893. Edited by GEORGE THOMAS JACKSON, M. D., secretary. Octavo, paper, pp. 82. New York. 1894.

This official report of the proceedings of this excellent association is well prepared, and contains some excellent illustrations. Much of the work has been already published in the *Journal of Cutaneous and Genito-Urinary Diseases*, hence need not be further commented upon at this time. A list of the publications of the members during the year ending September 1, 1893, is added for reference. The book should be bound in more substantial form.

---

### BOOKS RECEIVED.

Small Hospitals. Establishment and Maintenance. By A. Worcester, A. M., M. D., and Suggestions for Hospital Architecture with plans for a small hospital. By William Atkinson, Architect. Duodecimo, pp. vi.—114. Price, cloth, $1.25. New York : John Wiley & Sons, 53 East Tenth street. 1894.

Macrobiotic or Our Diseases and Our Remedies. For Practical Physicians and People of Culture ; by Julius Hensel, Physiological Chemist. Translated by Prof. Louis H. Tafel of Urbana University, O. From the second revised German edition. Octavo, pp. v.—201. Published by Boericke & Tafel, 1011 Arch street, Philadelphia.

Text-Book of Medical and Pharmaceutical Chemistry, By Elias H. Bartley, B. L., M. D., Professor of Chemistry and Toxicology in Long Island College Hospital ; Dean and Professor of Organic Chemistry in the Brooklyn College of Pharmacy, etc., etc. Third edition, revised and enlarged with eighty-four illustrations, Small 8vo, pp. xiv.—684. Price, $3.00. Philadelphia : P. Blakiston, Son & Co., 1012 Walnut street. 1894.

Index—Catalogue of the Library of the Surgeon-General's Office' United States Army. Authors and Subjects. Vol. XV., Universidad—Vzoroff. Quarto, pp. 842. Washington : Government Printing Office. 1894.

The Graphic History of the Fair. Containing a sketch of International Expositions, a review of the events leading to the discovery of America and a History of the World's Columbian Exposition held in the City of Chicago, State of Illinois, May 1 to October 31, 1893. Describing the notable features in the several departments, the sculpture of buildings and grounds, the department edifices, the state and foreign buildings and pavilions, the exhibits, etc., etc. With nearly 1,000 illustrations. Chicago : The Graphic Company, 358 Dearborn street. 1894.

Where to send Patients Abroad for Mineral and other Water Cures and Climatic Treatment. By Dr. Thomas Linn, Doctor of Medicine, Faculty of Paris ; Doctor of Medicine and Surgery, University of New York, etc., etc. Price, paper, 25 cents ; cloth, 50 cents. Physicians' Leisure Library. Detroit, Mich.: George S. Davis. 1894.

A Hand-Book of Medical Microscopy, for Students and General Practitioners, including chapters on Bacteriology, Neoplasms and Urinary Examinations. By James E. Reeves, M. D., author of A Practical Treatise on Enteric or Typhoid Fever. With a glossary and numerous illustrations (partly in colors). Price, $2.50, net. Pp. xv.—237. Philadelphia : P. Blakiston, Son & Co., 1012 Walnut street. 1894.

Nineteenth Annual Report of the Secretary of the Board of Health of the State of Michigan, for the fiscal year ending June 30, 1891. Pp. cxlvii.—346. Lansing : Robert Smith & Co., State Printers and Binders. 1894.

